# Supplementary material for: Maternal employment and child nutritional status in Uganda
Source: PLoS One. 2019 Dec 19;14(12):e0226720. doi: 10.1371/journal.pone.0226720 (PMC6922416; doi:10.1371/journal.pone.0226720)
Supplement: Attachment — Submitted filename: Reviewer Comments.docx [file pone.0226720.s001.docx]

This is an interesting paper presenting original research examining the relationship between maternal employment and child nutritional status. The paper needs some minor changes. Following are some comments and suggestions that the authors could consider:

1. One of my major concerns about the paper was that certain statements made in the discussion section do not match the results presented in Table 2. For e.g.:

a)in Pg 15 lines 327-329:

‘Related to studies elsewhere, this study showed that children whose mothers were engaged in  professional, technical and managerial work had lower odds of stunting and underweight  compared with other occupation categories .’

However, the results from table 2 show that children whose mothers were engaged in  professional, technical and managerial work had significantly lower odds of stunting  compared with agricultural and manual workers (not all other occupations), also results for underweight were not significant.

b) Line 392-393 (Pg 18) says ‘Female children showed reduced odds of stunting than male children’

This should be wasting and not stunting.

It would be great if the authors double check the results reported in the discussion section with the Table 2.

2. Could multiple children in the analyses have the same mother? Is this taken into account in the regression?

3. There should be a clear link between the second last paragraph and the last paragraph in the introduction section (pg 4, line 90-99) and Pg 5 line 101-102. Perhaps adding a paragraph or two on: more studies on maternal employment and child health, identifying gaps in the literature, explaining their contribution clearly, and highlighting that they are one of the first to examine the relationship between maternal employment, and child health outcomes in Uganda. Also, lines 101-106 (Pg 5) do not lead up to the research question, and could be omitted.

A few minor points:

4. Pg 3 lines 53-60, please give reference of the data source.

5.Pg 5: line 117: What is the full form of UGKR?

6.Minor clarification: Pg 7 line 156: refers to those recoded to 2?

7. In the discussion section the authors could consider linking study conclusion to other studies in Sub Saharan Africa that addressed similar questions. Some studies that the authors might find useful are:

Negash, C., Whiting, S. J., Henry, C. J., Belachew, T., & Hailemariam, T. G. (2015). Association between maternal and child nutritional status in Hula, rural Southern Ethiopia: A cross sectional study. PloS one, 10(11), e0142301.

Ukwuani, F. A., & Suchindran, C. M. (2003). Implications of women's work for child nutritional status in sub-Saharan Africa: a case study of Nigeria. Social Science & Medicine, 56(10), 2109-2121.

Maxwell, D. (1999). The political economy of urban food security in Sub-Saharan Africa. World Development, 27(11), 1939-1953.

8. Pg 18 line 386-387: reframe the sentence to say that ‘children born to older women have better health outcomes’ instead of the existing sentence that says ‘older women have better health outcomes than younger women’.
